# The Formation of Bivalents and the Control of Plant Meiotic Recombination

**DOI:** 10.3389/fpls.2021.717423

**Published:** 2021-09-07

**Authors:** Yared Gutiérrez Pinzón, José Kenyi González Kise, Patricia Rueda, Arnaud Ronceret

**Affiliations:** Laboratory of Cytogenomics of Meiosis, Instituto de Biotecnología, Departamento de Biología Molecular de Plantas, National Autonomous University of Mexico (UNAM), Cuernavaca, Mexico

**Keywords:** Meiosis, recombination, synapsis, obligate crossing-over, interference, CO homeostasis, heterochiasmy

## Abstract

During the first meiotic division, the segregation of homologous chromosomes depends on the physical association of the recombined homologous DNA molecules. The physical tension due to the sites of crossing-overs (COs) is essential for the meiotic spindle to segregate the connected homologous chromosomes to the opposite poles of the cell. This equilibrated partition of homologous chromosomes allows the first meiotic reductional division. Thus, the segregation of homologous chromosomes is dependent on their recombination. In this review, we will detail the recent advances in the knowledge of the mechanisms of recombination and bivalent formation in plants. In plants, the absence of meiotic checkpoints allows observation of subsequent meiotic events in absence of meiotic recombination or defective meiotic chromosomal axis formation such as univalent formation instead of bivalents. Recent discoveries, mainly made in Arabidopsis, rice, and maize, have highlighted the link between the machinery of double-strand break (DSB) formation and elements of the chromosomal axis. We will also discuss the implications of what we know about the mechanisms regulating the number and spacing of COs (obligate CO, CO homeostasis, and interference) in model and crop plants.

## Introduction

Meiosis is one of the most dynamic processes for a plant genome (Ronceret and Pawlowski, 2010; Prusicki et al., 2019). To achieve a reductional division, the meiotic cell goes through one round of DNA replication followed by two cell divisions (Mercier et al., 2015). The meiotic divisions have evolved from the machinery toolkit used by the regular mitotic division with additional regulatory functions allowing the reductional division (Wilkins and Holliday, 2009). Several differences between meiosis and mitosis are discernible already at prophase I with the introduction of meiotic-specific processes such as meiotic recombination, pairing, and synapsis of homologs. During the whole meiotic prophase I, the nuclear chromosome content is duplicated and each homolog is constituted by two sister chromatids. Bivalents are defined as connected homologous chromosomes, forming a unit of four DNA molecules, essential for the equilibrated segregation of the chromosome pool. The formation of bivalents occurs during the prophase I of meiosis and involves the coordination between homologous recombination, pairing, and synapsis (Mercier et al., 2015). During meiotic metaphase I, a specific bipolar conformation of the meiotic spindle attachment to centromeres allows the segregation of these recombined bivalents. In plants, male and female meiosis occur in different organs. Though most meiotic mechanisms are shared between sexes, some differences have long been observed between male and female meiotic recombination rates. After the two successive meiotic divisions, haploid spores, which contain only one set of each chromosome, are formed. These separated male or female spores undergo the gametophytic phase giving rise to distinct male and female gametes. Fertilization between gametes restores the diploid state crucial for the sexual life cycle and the genome maintenance of the species.

Understanding the formation of how new meiotic DNA molecules are formed is of special value for breeding since it is a fundamental basis for genetics, evolution, and genomic crop improvement (Melamed-Bessudo et al., 2016; Lambing et al., 2017; Blary and Jenczewski, 2019; Bolaños-Villegas and Argüello-Miranda, 2019; Taagen et al., 2020; Kuo et al., 2021).

**FIGURE 1:**
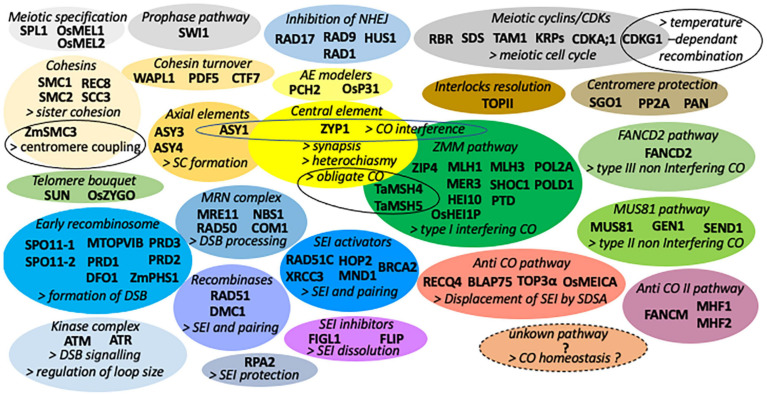
List of proteins regulating bivalent formation grouped by functional modules under a different colored bubble. For simplification, the list only gives the name of Arabidopsis proteins unless the two-letter symbol of the species is specified before the protein name (Os rice, Ta durum wheat, and Zm maize).

This review will focus on the recent advances in the understanding of the genetic control of meiotic recombination and bivalent formation in diploid plant species, mainly Arabidopsis, rice, and maize. For the more complex bivalent and multivalent formation in polyploid plants [Refer the reviews of Cifuentes et al. (2010), Mason and Wendel (2020), and Svačina et al. (2020)]. Several important discoveries have been made in these last few years concerning the mechanisms of crossing-over (CO) interference (impeding the formation of adjacent COs), non-crossing-over (NCO) pathways, obligate CO (to maintain at least one CO by bivalent), CO homeostasis [the buffering of CO numbers despite the reduction in double-strand breaks (DSBs) number], and heterochiasmy (difference in male and female CO frequencies). For an overview of the plant meiotic genes and mechanisms discovered before 2018, see the excellent reviews of Luo et al. (2014), Mercier et al. (2015), and Wang and Copenhaver (2018). An overview of the proteins regulating bivalent formation grouped by functional modules is listed in Figure 1.

## Regulation of the Meiotic Cell Fate and Meiotic Transcriptome

In plants, the germline fate acquisition where meiosis will occur involves the specific transcription factor SPOROCYTELESS also known as NOZZLE in Arabidopsis (Yang et al., 1999; Wei et al., 2015) and rice (Ren et al., 2018). In rice, the ARGONAUTE protein MEL1 plays an essential role in male and female meiotic cell fate (Nonomura et al., 2007; Komiya et al., 2014; Liu and Nonomura, 2016). The rice MEL2 is an RNA recognition motif protein binding the 3′-UTRs and involved in the translational regulation of key meiotic genes (Nonomura et al., 2011; Miyazaki et al., 2015). In Arabidopsis, no MEL orthologs have been described but AGO9 and AGO4 are involved in female gamete specification (Olmedo-Monfil et al., 2010), while small interfering RNA inhibits retrotranspositions in the male germline (Long et al., 2021). Argonautes are the key players in distinct small RNAs (sRNAs) pathways involved in transcriptome regulation (Oliver et al., 2014). Transcriptomic analysis of different steps of germline cells and meiocytes has revealed dramatic transcriptomic changes during prophase I in various plant species. In maize, single-cell RNA sequencing reveals a profound two steps reorganization of the transcriptome at the leptotene stage when meiotic recombination initiate (Nelms and Walbot, 2019). The sRNAs (micro-RNA and phased secondary small interfering RNA) are particular dynamics during prophase in rice, maize, sunflower, soybean, and cucumber (Dukowic-Schulze et al., 2016; Flórez-Zapata et al., 2016; Huang et al., 2020; Zhang et al., 2021) and, at least in Arabidopsis male meiocytes, play a critical role in meiotic recombination potentially *via* the AGO4 pathway (Oliver et al., 2016, 2017; Pradillo and Santos, 2018).

Though sex-specific transcriptomes have been obtained in various plant species (Dukowic-Schulze et al., 2020; Liu et al., 2020; Barakate et al., 2021), a systematic comparison between male and female meiotic transcriptomes has not yet been performed.

In maize, hypoxia arising naturally within growing anther tissue was reported to act as a positional cue to set male germ cell fate (Kelliher and Walbot, 2012). The cytology of plant female meiosis has been historically more challenging but is now prone to analysis due to new techniques of the whole immunolocalization of plant ovules (Escobar-Guzman et al., 2015; Gordillo et al., 2020). In Arabidopsis, the specification of only one germ cell line per ovule has been analyzed and involved complex positional clues and identify RBR1 as a central hub for female meiocyte differentiation (Zhao et al., 2017). RBR1 is also required for the recombinase RAD51 localization to DNA lesions (Biedermann et al., 2017). *SWITCH1/AMEIOTIC* is an essential gene allowing the entry in male and female meiosis. SWI1 was recently identified as a functional Sororin-like antagonist (Yang et al., 2019) of the WINGS APART-LIKE (WAPL) that removes cohesin from chromosome *via* the prophase pathway before the action of separase at anaphase onset (De et al., 2014). Accordingly, SWI1/AM1 is a nuclear protein installed on the whole chromatin from premeiotic replication and is only maintained in centromere regions during pachytene in maize (Pawlowski et al., 2009). However, this remaining centromeric localization was not observed in rice (Che et al., 2011) suggesting plant-specific variations for this regulation.

## Formation of the Meiotic Cohesin Complex

DNA replication is followed by the appearance of meiotic-specific components of the cohesin complex (formed by SMC1, SMC3, SCC2, SCC3, and REC8) (Cai et al., 2003; Chelysheva et al., 2005; Wang et al., 2020). Cohesin turnover and localization on chromatin are mediated by WAPL (De et al., 2014), PDS5 (Pradillo et al., 2015), and CTF7 (Singh et al., 2013). REC8 is a meiotic-specific kleisin that replaces the mitotic SCC1 subunit in this complex. It was shown in tomato that the four subunits of the meiotic cohesin complex are discontinuously distributed along the chromosome length from leptotene through early diplotene (Qiao et al., 2011). In maize, SMC3 is essential for sister chromatid cohesion and facilitates centromere coupling (Zhang et al., 2020d), a peculiar configuration associating the centromeres before pairing commences along chromosome arms (Ronceret et al., 2009). The current working models of chromatin organization suppose that the meiotic cohesin complex forms a ring attaching the two replicated sister chromatids and organizes the chromatin by forming the base of chromatin loops (Zickler and Kleckner, 2015; Kim and Choi, 2019; Grey and de Massy, 2021). Chromosome conformation capture (HiC) experiments have not yet been performed on plant meiotic genomes to analyze their chromosomal loop domain organization (Golicz et al., 2020). In Arabidopsis, ChIP-seq experiments have shown that REC8 is associated with repetitive centromeric and pericentromeric regions of high nucleosome occupancy, the opposite of where meiotic DSBs and crossovers are found at the chromosome and fine scales (Lambing et al., 2020b). REC8 containing cohesin complex is largely protected by the Shugoshin (SGO)-PP2A complex around centromeres during meiosis I (Zamariola et al., 2014; Yuan et al., 2018; Zhang et al., 2019) and by PAN during interkinesis (Cromer et al., 2019). These protections allow the coordinated separation of the homologous chromosomes during meiosis I and the release of the sister chromatin only during meiosis II *via* a separase-dependent proteolytic cleavage of the centromeric kleisin subunit REC8 (Cromer et al., 2019).

## Formation of the Axial Element of the Synaptonemal Complex

The synaptonemal complex (SC) is a proteinaceous ultrastructure formed of two axial elements (AEs) and a central element. Elements of the AEs are installed on chromatin during leptotene before synapsis occurs. In plants, several components of the AEs have been identified: the HORMA domain ASY1/PAIR2 (Armstrong et al., 2002; Nonomura et al., 2004a), the associated coiled-coiled proteins ASY3/DSY2/PAIR3 (Yuan et al., 2009; Ferdous et al., 2012; Lee et al., 2015; Osman et al., 2018) and ASY4 (Chambon et al., 2018). The cohesin REC8 is supposed to anchor chromatin to the AEs of the SC *via* PAIR3/ASY3 (Wang et al., 2011) though a direct interaction between REC8 and any AE protein is yet unknown. However, in Arabidopsis, ASY1 and REC8 ChiP-seq strongly correlate suggesting that both proteins associate with similar regions of the genome at global and fine scales (Lambing et al., 2020b). In maize, DSY2 (homolog of ASY3 and PAIR3) forms an alternative pattern with ASY1 on AE and can interact with ZYP1 while ASY1 cannot (Lee et al., 2015). In addition, the AAA^+^ ATPase PACHYTENE CHECKPOINT 2 (PCH2)/CRC1 is essential for the ASY1 depletion before synapsis (Lambing et al., 2015). Its interacting partner P31^comet^ participates in ASY1 import in the nucleus and the removal of non-phosphorylated ASY1 from the chromosomal axis (Balboni et al., 2020). In rice, CRC1/PCH2 can directly interact with ZEP1/ZYP1 while P31 cannot (Ji et al., 2016). In addition, the role of some SC proteins in the initiation of meiotic recombination such as DSY2/ASY3 in maize (Lee et al., 2015), CRC1/PCH2 (Miao et al., 2013) and P31 in rice (Ji et al., 2016) pinpoints to the essential role of AE elements on the initiation of meiotic recombination.

## Formation of the Early Recombinosome

During meiosis, two homologous DNA molecules can form new recombined ones using the general mechanics of the error-proof DNA damage repair pathways (Mercier et al., 2015; Wang and Copenhaver, 2018; Zelkowski et al., 2019). The initiation of meiotic recombination starts with the introduction of DNA DSBs. The DSBs are formed by the SPO11 complex composed of the catalytical A subunits, SPO11-1 (Grelon et al., 2001; Edlinger and Schlögelhofer, 2011; Da Ines et al., 2020) and SPO11-2 (Stacey et al., 2006; Hartung et al., 2007b; Benyahya et al., 2020), associated with two B subunits, called MTOPVIB of the class II topoisomerase type VI (Fu et al., 2016; Vrielynck et al., 2016; Xue et al., 2016). The Arabidopsis *spo11-1* mutant has an interesting distinctive 23 and 24 nt sRNA profile than wild type in male meiocytes. These SPO11-1-dependent sRNAs are mapped to bind coding sequences and some CO feature motifs, while some sRNAs can target some meiotic genes such as RAD51 or ASK1 (Huang et al., 2019). Whether or not these sRNAs are produced at or near the site of DSBs and represent a signaling mode for repairing the DSB requires further work.

Though every CO is derived from a DSB event, not all DSBs are repaired as COs, and a vast majority of DSBs result in NCOs (Mercier et al., 2015). In maize, the CML228 inbred line that has naturally less DSBs (evaluated by the RAD51 number) has a correlated decreased CO number compare to B73 and other inbreds, indicating that the level of CO homeostasis is limited (Sidhu et al., 2015). In Arabidopsis, hypomorphic *spo11-1* mutants that reduce the DSB number also diminish the CO number but interestingly also alter the pattern of CO toward the telomeres (Xue et al., 2018). These data suggest that, at least in these two model plant species, CO homeostasis is not observed or limited.

In maize, using superresolution microscopy, it was observed that only a subset of SPO11-1 foci, the one closely associated with the AEs, correspond to the number of DSBs formed in leptotene (Ku et al., 2020). This suggests that the topoisomerase II-like nuclease function of the SPO11-1 complex occurs only when it is associate with the AE (Ku et al., 2020). Whether or not this particular configuration allows the nuclease activity of only one of the two attached sister chromatids is not known. The SPO11-1 “early recombinosome” complex also contains various accessory proteins that might participate in the tethering between the chromatin loop and the axis where DSBs are formed. SPO11-1 and MTOPVIB can interact with PRD1 (Vrielynck et al., 2016; Tang et al., 2017). In addition, rice and maize MTOPVIB, rice PRD1, and Arabidopsis PRD2/MPS1 are required for the assembly of the meiotic bipolar spindle (Ji et al., 2016; Xue et al., 2019; Jing et al., 2020; Shi et al., 2021). It was long recognized in maize that the meiotic spindle can associate around chromatin independently of the formation of the bivalent (Chan and Cande, 1998; Nannas et al., 2016) suggesting that the multipolar spindle observed in the meiotic recombination mutants might be a consequence of the formation of univalents instead of bivalents. The rice PRD1 initially forms numerous foci during leptotene, progressively restricted to few foci colocalizing with centromeric CENH3 and other kinetochore proteins (MIS12, NDC80, and CENP-C) at pachytene (Shi et al., 2021). PRD1 can directly interact with REC8 and SGO1 (Shi et al., 2021) suggesting a mechanism for early coordination between DSB formation and meiotic spindle organization. Other SPO11-associated factors such as DFO1, PRD1, PRD2, and PRD3/PAIR1 have also been identified as essential for DSB formation (Nonomura et al., 2004b; De Muyt et al., 2007, 2009; Zhang et al., 2012) but their relationship, specific function, and potential interaction with the cohesin and AE proteins still need to be explored (Mercier et al., 2015; Kim and Choi, 2019). PHS1, discovered in maize (Pawlowski et al., 2004), is poorly conserved with the rec114 yeast DSB factor but has a divergent function in plants. It is involved in the nuclear localization of the RAD50 protein into the nucleus in maize and Arabidopsis (Ronceret et al., 2009). SKI8 is also not conserved between yeast and Arabidopsis (Jolivet et al., 2006). Interestingly, the PRD2/MPS1 can form different splicing isoforms depending on the methylation status of its intron 9, dependent on the RNA-directed DNA methylation pathway (Walker et al., 2018).

## Genomic Mapping of DSBs and Recombination Motifs

Several studies using ChIP or SPO11 oligonucleotide sequencing have now revealed the genomic pattern of DSBs in maize and Arabidopsis. As previously well-known, genomes contain hotspots of COs, that are now correlated with genomic hotspots regions more prone to form DSBs (He et al., 2017; Choi et al., 2018; Tock and Henderson, 2018). In these two diploid species, DSBs are associated with specific active chromosome features such as transcriptional start sites that are depleted of nucleosomes. In Arabidopsis, several motifs associated with recombination have been defined. The A-rich motif is preferentially associated with promoters, while the CCN repeat and the CTT repeat motifs are preferentially associated with genes (Shilo et al., 2015). The motifs correlating with COs are not necessarily identical to motifs correlating with sites of DSB. This suggests that several genomic contexts required for the different steps of recombination progressively shape the choice of chromosomal exchange sites. The presence of a similar but not identical 20-bp-long GC-rich degenerate DNA sequence motif was correlated with DSB formation in maize and Arabidopsis (He et al., 2017; Choi et al., 2018). Interestingly, while DSBs are also formed in regions that will not form CO such as centromeric regions and repetitive DNA (especially RNA transposons), it was found that only DSBs formed in genic regions will form CO in maize (He et al., 2017). In Arabidopsis, mutants affecting the methylation of H3K9me2 and DNA CG and non-CG in the transposon-rich pericentromeric heterochromatin also increase the formation of DSB near centromeres (Underwood et al., 2018; Fernandes et al., 2019). In maize, the *mop1* mutation (homolog of RDR2) that removes CHH methylation adjacent to hotspots also affects the recombination landscape, increasing it in the chromosome arm but decreasing it in pericentromeric regions (Zhao et al., 2021). These data indicate that though the molecular bases are distinct in species of different genome sizes, and with relative repetitive element contents, a strong effect of epigenetic and chromatin state controls the fate of the early meiotic recombinosome.

## Signaling and Processing of the DSBs

The programmed DSBs are identified by the signaling pathway of DNA damages *via* the ATM and ATR kinases (Kim and Choi, 2019; Zhang et al., 2020a). In budding yeast, ATM/ATR can phosphorylate REC8 and other chromosome axis proteins and, therefore, modulate CO homeostasis (Kim and Choi, 2019). It is not clear, however, if this is also the case in plants. The meiocyte nuclei use prepared recombination machinery to repair the numerous endogenous DSBs using a preferential homologous recombination pathway. The somatically preferred non-homologous end joining (NHEJ) is suppressed by the exonuclease (RAD9-RAD1-HUS1) 9-1-1 complex system (Che et al., 2014; Hu et al., 2016). This 9-1-1 complex itself is possibly recruited by the DNA damage sensor RAD17 (Hu et al., 2018). This inhibition is essential to avoid inaccurate interactions between non-homologous chromosomes during meiosis (Che et al., 2014). The DSBs are rapidly associated with the phosphorylation of histone H2AX and processed by endonuclease and exonucleases activities of the MRN complex composed of MRE11, RAD50, NBS1, and COM1 (Bleuyard et al., 2004; Puizina et al., 2004; Uanschou et al., 2007; Waterworth et al., 2007; Lohmiller et al., 2008; Šamanić et al., 2013; Wang Y. et al., 2018) creating 5′ overhang sequences. These sequences are recognized by specialized single DNA strand affine replication protein A (RPA) types such as RPA1a in Arabidopsis (Osman et al., 2009) or RPA1C and RPA2C in rice (Li et al., 2013). RPAs are generally involved in telomere-length maintenance (Aklilu et al., 2020) suggesting a functionalization of this single DNA strand capping for the processing of meiotic DSBs.

## Single-End Invasions and Pairing

These single ends can invade homologous sequences (process called single-end invasion or SEI) thanks to the recombinase activities of RAD51 and DMC1 (Couteau et al., 1999; Li et al., 2004). These recombinases have the properties to form a strong homology-based DNA triple helix called a displacement loop (D-loop) (Kurzbauer et al., 2012; Wang et al., 2016; Singh et al., 2017; Colas et al., 2019; Draeger et al., 2020). The RAD51 and DMC1 are part of the same recombinase protein family also containing RAD51C and XRCC3 that derive from the same ancestor but have undergone subfunctionalization (Bleuyard et al., 2004; Da Ines et al., 2013; Pradillo et al., 2014). RAD51C and XRCC3 facilitate the RAD51 chromosome localization (Su et al., 2017; Jing et al., 2019; Zhang et al., 2020c). RAD51 and DMC1 also associate with other proteins modulating their activities such as BRCA2 (Siaud et al., 2004; Dray et al., 2006; Seeliger et al., 2012; Fu et al., 2020), FIGL1 and FLIP (Zhang P. et al., 2017; Fernandes et al., 2018a; Kumar et al., 2019), or MND1, and HOP2 (Vignard et al., 2007; Uanschou et al., 2013; Aklilu et al., 2020). Rice MND1/MSF1 can interact with RPA2b and HOP2 (Lu et al., 2020), while OsHOP2 can directly interact with the SC central element ZEP1/ZYP1 suggesting a second mechanism for the link observed between early recombination and synapsis (Shi et al., 2019) at the SEI step. The excess number of SEI creating several interconnections between homologous chromosomes (also called interhomolog joint molecules) were proposed to be involved in the pairing process (Pawlowski et al., 2003). Pairing allows the recognition between accurate homologs before its stabilization by the polymerization and lateral extension of the SC during zygotene.

## Anticrossover Pathways

The number of DSB and SEI is far greater than the number of COs and the vast majority of DSBs [around 85% in Arabidopsis (Higgins et al., 2004) and 97.7% in durum wheat (Desjardins et al., 2020)] are resolved as NCOs. NCOs are formed when the SEI occurs on the sister chromatid but also when a D-loop formed on the homologous chromosome is resolved in a configuration that only involves the exchange of genetic material in a short sequence called conversion tracts (Mercier et al., 2015; Wang and Copenhaver, 2018). Three parallel anti-CO pathways have been discovered using suppressor genetic screens of *zmm* meiotic recombination mutants in Arabidopsis (Séguéla-Arnaud et al., 2015).

The first NCO pathway involves the SGS1/BLM helicases homologs RECQ4A and RECQB (Hartung et al., 2007a; Higgins et al., 2011) and the topoisomerases TOP3α (Hartung et al., 2008) associated with BLAP75/RMI (Chelysheva et al., 2008), which unwinds D-loops leading to a sixfold CO number increase in Arabidopsis (Séguéla-Arnaud et al., 2015). In rice, the MEICA protein that interacts with TOP3a also has an anticrossover activity (Hu et al., 2017).

The anti-CO pathway that involves the FANCM helicase possibly displaces the invading strand through the synthesis-dependent strand annealing (SDSA) process (Crismani et al., 2012). SDSA can form NCOs by annealing the SEI with the other end of the DSB, repairing the DSB using the original DNA molecules. FANCM has two binding cofactors MHF1 and MHF2 that also limit the number of COs formed *via* the type II non-interfering pathway (Dangel et al., 2014; Girard et al., 2014). Interestingly, the anti-CO effect due to FANCM is more pronounced in inbred than in hybrids backgrounds (Girard et al., 2015). The FANCM pathway also affects COs in a *Brassica rapa* pure line (Blary et al., 2018). In lettuce, the *fancm* mutant shows a univalent phenotype not observed in other species (Li et al., 2021) indicating possible divergence in the regulation of this pathway or different consequences between species of different genome sizes.

Another cumulative NCO pathway involves the FIDGETIN AAA-ATPase FIGL1 (Girard et al., 2015; Zhang P. et al., 2017) and its partner FLIP (Fernandes et al., 2018a). FIGL1 directly interacts with RAD51 and DMC1 and is proposed to limit SEI and CO number (Fernandes et al., 2018a). Using the mutants of these different pathways or combining them together or with elevated expression of the procrossover factors HEI10 (Serra et al., 2018) are particularly interesting for agronomy since it allows to unleash the number of CO by several folds in various plant species and potentially speed up new breeding strategies (Fernandes et al., 2018b; Mieulet et al., 2018). Interestingly, these CO increase do not cause problems in chromosome segregation. The relative role of these different pathways in male vs. female plant meiosis requires further analysis.

## Synapsis and Role of the SC in the Regulation of Recombination

The central element ZYP1 of the SC starts polymerizing during zygotene to form a protein complex resembling a zipper structure connecting the two meiotic homologous chromosomes from telomere to telomere at pachytene. The two AEs are called lateral elements once they form this tripartite structure (Higgins et al., 2005; West et al., 2019; Darrier et al., 2020; Kurzbauer et al., 2021). Although two redundant ZYP1 proteins sharing 87% identity are present in Arabidopsis; their relative role is still unknown. The SC can regulate the number and spacing of CO. While barley *zyp1* mutants show limited CO numbers (Barakate et al., 2014), the homolog rice *zep1* mutants have the opposite effect (Wang et al., 2010). The SC components have diverged rapidly among eukaryotes but the general SC structure is conserved (West et al., 2019). These results might reflect divergent modes of regulation of the SC on CO between different species. ZYP1 was also recently reported as required for CO interference and the obligate CO (France et al., 2021). In Arabidopsis *zyp1a/b* null mutant, heterochiasmy is abolished (Capilla-Pérez et al., 2021). These recent data suggest that the SC coordinates the regulation of obligate CO, interference, and heterochiasmy. Arabidopsis *asy1* mutants also abolish CO interference (Lambing et al., 2020a). ASY1 acts as an antagonist of telomere-led recombination in a gene dosage-sensitive manner (Lambing et al., 2020a). The ASY1 immunolocalization signal disappears concomitantly with the loading of the central element ZYP1 (Lee et al., 2015). This suggests that obligate CO, heterochiasmy, and interference mechanisms are not directly mediated by either ASY1 or ZYP1 but rather involve the regulation of the SC length. ASY1 can be phosphorylated by CDKA;1 counteracting the ASY1 disassembly activity of PCH2 and P31 (Yang et al., 2020), which suggests a dynamic control of SC length regulation. The phosphorylation of the ASY1 protein increases its binding affinity with the chromatin-anchoring ASY3/DSY2 protein (Yang et al., 2020). ATM is another meiotic protein kinase essential to limit DSB number; it regulates chromatin loop size and affects SC length and width (Kurzbauer et al., 2021). The relative role of ATM and CDKA1;1 in the phosphorylation of SC components or other meiotic proteins is still unknown.

## Formation of the Late Recombinosome and Crossover Pathways

The DNA repair of the damaged molecules involves the formation of double Holliday junctions (dHJs) that are resolved by resolvases leading to CO or NCO. Two pathways of CO formation have been described in plants.

The interfering pathway (CO type I) positions CO with non-random spacing between each CO event. In general, the CO type I accounts for the majority (80–95%) of all COs in plant species (Mercier et al., 2015). It involves the ZMM pathway, namely, MER3 (Chen et al., 2005; Mercier et al., 2005), ZIP4 (Chelysheva et al., 2007), the DNA mismatch repair mutS/mutL homologs MSH/MLH (MSH4, MSH5, MSH7, MLH1, and MLH3) (Higgins et al., 2004, 2008b; Chelysheva et al., 2010; Colas et al., 2016) and HEI10 (Chelysheva et al., 2012; Ziolkowski et al., 2017). In rice, a new member of the ZMM pathway was discovered through its interaction with HEI10, MSH4, and ZIP4 and named HEI1P1 (Li et al., 2018). SHOC1 and PTD, which were described in Arabidopsis as involved in the type I CO pathway (Macaisne et al., 2008, 2011), are conserved and play similar roles in rice (Ren et al., 2019). Interestingly, it was found that the obligate COs (that ensure the correct chromosome segregation during anaphase I) are maintained by MSH4 and MSH5 in durum wheat (Desjardins et al., 2020). In the allotetraploid *Brassica napus*, reducing the MSH4 copy number prevents non-homologous CO (Gonzalo et al., 2019). The analysis of hypomorph mutants of two essential B-class DNA polymerases (the delta POLD1 supposed to be involved in DNA lagging strand synthesis and the Epsilon POL2A thought to be involved in DNA leading strand synthesis) has shown that they are also involved in the formation of type I COs (Huang et al., 2015; Wang C. et al., 2018). It was first hypothesized that elongation activity of these polymerases is required for the process of meiotic recombination but the multifunctionality of these POL proteins, containing exonuclease proofreading domains (Ronceret et al., 2005), could complicate the interpretation of the activity required during meiotic recombination. In addition, DNA polymerases are involved in the deposition of the H3K4me3 transcriptionally active epigenetic marks linked to DSB formation and participate in DNA repair (Yin et al., 2009; Iglesias et al., 2015) suggesting other possibilities for the role of DNA POL in the formation of type I COs. However, since most of the *pol* mutants have embryo-lethality phenotypes (Ronceret et al., 2005; Wang et al., 2019) this is a difficult topic to study.

The second minor CO pathway (type II or non-interfering) can form closely spaced COs. In plants, it involves the dHJ resolvases (structure-specific endonuclease) MUS81 (Higgins et al., 2008a), GEN1 (Wang et al., 2017), and SEND1, which is also essential for telomere stability (Geuting et al., 2009; Olivier et al., 2015).

Though it was found to be involved in the mechanism of interference in yeast, the topoisomerase TOPII was not found to have an effect on CO interference in Arabidopsis but to facilitate interlocks resolution (remove interlacement of different bivalents at the time of synapsis) (Martinez-Garcia et al., 2018) that are normally all resolved by pachytene (Wang et al., 2009). Interestingly, TOPII is associated with the chromosome axis and accumulates in entangled regions during the zygotene stage (Martinez-Garcia et al., 2018). In Arabidopsis, a second non-interfering pathway of CO (called type III non-interfering CO in Figure 1), parallel to the MUS81 (type II) CO pathway, depends on FANCD2 and contributes to the formation of some non-interfering COs (Kurzbauer et al., 2018). The hotspots and coldspots of recombination are supposably due to the combined effects of chromatin features and the different anticrossover and crossover pathways. The relative mechanisms by which the CO rate is modulated at these sites still require further exploration.

## Effects of Genomic Regions, Centromere Pairing, Telomere Bouquet, and Repeated DNA Recombination

Various genomic regions are known to have variable recombination rates in various plant species. New whole genome sequencing techniques have now given us a clear vision of the recombination maps at a fine scale in several plant species (Hellsten et al., 2013; Luo et al., 2019; Rowan et al., 2019). It is known that the genomic recombination rate is influenced by epigenetic marks, the genetic background (Sidhu et al., 2015; Ziolkowski et al., 2017; Dreissig et al., 2019; Lawrence et al., 2019), and the level of heterozygosity (Ziolkowski et al., 2015). It also greatly depends on chromatin structural variation where large inversion and translocations can suppress recombination (Rowan et al., 2019; Termolino et al., 2019). The molecular basis of this suppression is still unclear but probably involves the abnormal SC installation on unpaired chromatin loop domains.

Chromosome conformation changes are highly dynamic during meiotic prophase, involving active mechanisms to gather telomeres at the nuclear envelope (called telomere bouquet), centromere coupling, and chromosome pairing and synapsis (Sepsi and Schwarzacher, 2020; Lenykó-Thegze et al., 2021).

In maize and rice, the SUN proteins are involved in telomere bouquet formation (Murphy et al., 2014), synapsis, and CO formation (Zhang et al., 2020b). SUN1 and SUN2 Arabidopsis mutants delay the progression of meiosis, affect synapsis, and reduce the chiasma number (Varas et al., 2015). The role of AtSUN1 and AtSUN2 on the bouquet has not yet been analyzed since the Arabidopsis telomere bouquet was only recently defined using techniques that maintain the 3D structure of the nucleus intact (Hurel et al., 2018). In rice, the bouquet is dependent on the PAIR3/ASY3 AE element (Wang et al., 2011) and on the F-Box ZYGO protein that also affects the initiation of homologous pairing (Zhang F. et al., 2017). In maize, SPO11-1 foci are transiently observed on the nuclear periphery and seem excluded from the nucleolus (Ku et al., 2020) suggesting a potential gathering of the DSBs machinery at the nuclear envelope and that its chromatin localization is not homogenous on the genome. Interestingly in Arabidopsis, the repetitive nucleolus organizing regions (NORs) acquired distinct chromatin characteristics during meiosis with strong ASY1 signals and the absence of the synaptic ZYP1 protein. The nucleolus employs an NHEJ mechanism requiring LIG4 (instead of the homologous recombination pathway dependent on RAD51) to repair the few DSBs produced in NORs and avoid unequal recombination in the repetitive recombinant DNA clusters (Sims et al., 2019). The presence of fewer COs in the heterochromatic repetitive knob region was observed cytogenetically in maize male meiocytes (Stack et al., 2017). However, by contrast to the nucleolus, knob meiotic recombination still uses the homologous recombination pathway as observed by the presence of MLH1 foci. This indicates that the diminution of meiotic recombination in distinctive heterochromatin regions probably uses several distinct mechanisms.

## Effects of Age and Sex on Meiotic Recombination

A moderate effect of the age of the shoot apical meristem on the number of CO was reported in Arabidopsis (Francis et al., 2007; Toyota et al., 2011; Li et al., 2017; Saini et al., 2020). Whether or not these age effects also occur in other plants is still unknown.

Sex difference in CO frequency is called heterochiasmy. In Arabidopsis, the CO number is higher in male meiocytes than in female meiocytes (Drouaud et al., 2007; Giraut et al., 2011; Saini et al., 2020). By sequencing Arabidopsis male and female backcrossed plants, 4.58 crossovers were found in male backcrossed compared to 3.08 in female backcrosses (Capilla-Pérez et al., 2021), noting that only half of the true CO number can be identified since gametes inherit a single chromatid and CO involves only two of the four chromatids of a bivalent. In Arabidopsis and maize, the difference is attributed to the length of the SC and the distribution of CO is also different in male and female meiocytes (Giraut et al., 2011; Kianian et al., 2018; Lloyd and Jenczewski, 2019; Luo et al., 2019). It was recently demonstrated that heterochiasmy is enforced in Arabidopsis by the SC central element ZYP1 (Capilla-Pérez et al., 2021). This suggests that heterochiasmy and SC length differences in male and female meiocytes are regulated by a common molecular pathway.

## Effect of Environmental Conditions on Meiosis

In plants, meiosis occurs in flowers whose development was initiated *via* various past and present environmental clues (Antoniou-Kourounioti et al., 2021). The temperature variation is also known to modify the meiotic and somatic recombination rate using fluorescent-tagged lines (Francis et al., 2007; Li et al., 2017; Saini et al., 2017) correlated with cytological MLH1 foci counting (Lloyd et al., 2018). In Arabidopsis, both high (28°C) and low (8°C) temperature conditions increase meiotic recombination compared to medium temperature (18°C). Interestingly, external temperatures are negatively correlated with the SC length that is itself correlated with the CO number. A correlation between SC length and CO number per chromosome was found (von Wettstein et al., 1984). Consequently, the longer SC length observed at low temperatures can explain the higher number of CO but not the increase of CO number observed at the higher temperatures. This increase in CO due to high temperature is not due to an increase in DSB formation as observed with γH2A.X and RAD51 foci. These extra COs are class I (ZMM) pathway as evidenced by increased MLH1 and HEI10 focus numbers in male meiocytes (Blary et al., 2018). Using mutants of different CO pathways in Arabidopsis, it was confirmed that the extra COs are derived from the interfering type I CO pathways and not to the type II (Modliszewski et al., 2018; De Storme and Geelen, 2020). The response of CO number to external temperature is not a universal stress response since saline stress does not affect it. Though the effect of temperature on COs was also observed and analyzed in barley, it seems that the mechanism of action is distinct. In contrast to Arabidopsis, the SC length in barley male meiocytes increases with higher temperature. The number of CO type I is not altered but their position shifted toward more internal non-telomeric regions as observed with cytologically mapped MLH3 foci (Phillips et al., 2015). The same effect of the position shifting from distal to more internal CO is also observed for some chromosome arms in wheat (Coulton et al., 2020). Though it seems an attractive and easy parameter that could modulate CO in crops, it appears that extremal temperatures have also other deleterious effects on the progression of meiosis such as defects of secondary division and wall formation reducing euploidy and seed set (De Storme et al., 2012; Draeger and Moore, 2017; De Storme and Geelen, 2020).

The presence of the histone H2A.Z was determined as the marker of the thermosensory response in Arabidopsis, with H2A.Z deposition decreasing with increasing temperatures (Kumar and Wigge, 2010). In addition, the CO sites overlap with the presence of H2A.Z nucleosome at gene promoters (Choi et al., 2013). Arabidopsis mutants of the H2A.Z placement show lower CO frequency. The correlation between H2A.Z and CO frequencies could explain the part of the effect of lower temperatures increasing CO frequency but not the effects of higher temperatures. Indeed, the higher CO frequency of Arabidopsis plants grown at 12°C compared to plants grown at 21°C disappears in mutants defective for H2A.Z deposition (Kumar and Wigge, 2010). The relation between the deposition of H2A.Z and the phosphorylation of γH2AX associated with the formation of DSB is currently unknown.

Another key factor of this regulation of the meiotic recombination by temperature is the cyclin-dependent kinase CDKG1. Arabidopsis *cdkg1* mutants show temperature-sensitive meiotic defect at 23°C but not at 12°C with abnormally formed SC, lower CO frequency, and reduce the bivalent number (Zheng et al., 2014; Nibau et al., 2020b). There are temperature-dependent isoforms of CDKG1 (Nibau et al., 2020a). These isoforms can interact with the spliceosome and can regulate the splicing of other spliceosome components and the Callose synthase5 forming the pollen wall (Huang et al., 2013; Cavallari et al., 2018). It is still not known that whether or not the CDKG1-dependent temperature-sensitive regulation affects the production of different splicing variants of meiotic genes or affects H2A.Z deposition.

Another meiotic cyclin CDKA;1 has an important role in the regulation of the recombination landscape (Wijnker et al., 2019). CDKA;1 is also involved earlier in the germline fate decision *via* the inactivation of RBR1 (Chen et al., 2011; Zhao et al., 2017) pointing out the coordinating role of a peculiar meiotic CDK as a key factor for the meiotic fate and the regulation of meiotic recombination. What are the relative roles of the meiotic CDKs, the associated meiotic cyclins (such as SDS and TAM), and CDK inhibitors (KRPs) in the coordinated control of meiotic recombination in different temperature conditions remain to be analyzed.

Other factors such as climate, agrochemicals, heavy metals, combustible gasses, pharmaceuticals, and pathogens are known to modify meiosis in plants (Modliszewski and Copenhaver, 2017; Fuchs et al., 2018; Dreissig et al., 2019) but their mechanistic modes of action still need to be explored.

## Conclusion

The understanding of several fundamental meiotic processes has strongly advanced during the past few years thanks to many studies in model and non-model plant species. Figure 1 summarizes the different proteins and functional modules known to be involved in the formation of bivalents.

The new techniques of isolated cell high throughput sequencing will revolutionize the questions we can ask about the dynamic meiotic chromosome conformation through prophase I.

Though controversial for many years, the divergence of several basic molecular meiotic mechanisms is now clear between different plant species. Achiasmatic inverted meiosis has also been reported in few non-model plants (Cabral et al., 2014; Heckmann et al., 2014; Hofstatter et al., 2021) underlining the extreme diversity of the plant meiotic programs. It contradicts the predictive expected assumptions based on phylogenetic relationships between plant species. In this perspective, one of the future challenges will be to identify the actual biochemical functions of the meiotic proteins not only based on the putative function supposed by the homology of conserved protein families. These interspecific differences are probably the real essence of the meiotic process that has evolved to bring genomic diversity. Even in the same species, there are known sex and cell to cell variability (Wang et al., 2019). It underlines the importance of studying directly meiosis in crops to manipulate it properly. Increasing our meiotic manipulation tools for improving plant breeding strategies is essential to cope with the challenge of feeding 10 billon humans by 2050.

## Author Contributions

YGP and AR prepared and wrote the manuscript. JKGK and PR contributed to the survey of bibliographic references. All authors read and approved the final manuscript.

## Conflict of Interest

The authors declare that the research was conducted in the absence of any commercial or financial relationships that could be construed as a potential conflict of interest.

## Publisher’s Note

All claims expressed in this article are solely those of the authors and do not necessarily represent those of their affiliated organizations, or those of the publisher, the editors and the reviewers. Any product that may be evaluated in this article, or claim that may be made by its manufacturer, is not guaranteed or endorsed by the publisher.
